# Effects of the tidal dehydration stress on epiphytic bacterial community of the intertidal macroalga *Sargassum thunbergii*

**DOI:** 10.1128/spectrum.01948-25

**Published:** 2025-12-26

**Authors:** Bing Sun, Tao Sun, Kang Ji, Zhibo Yang, Jing Wang, Yayun Zhao, Xinlong Yu, Xuexi Tang, Hui Xiao

**Affiliations:** 1 MoE Key Laboratory of Evolution & Marine Biodiversity, College of Marine Life Sciences, Ocean University of China 12591, Qingdao, China; 2 Laboratory for Marine Ecology and Environmental Science, Qingdao Marine Science and Technology Center 554912 , Qingdao, China; 3 Qingdao Branch CCCC Water Transportation Consultants Co., LTD, Qingdao, China; 4 North China Sea Environment Monitoring Center, State Oceanic Administration 646264 , Qingdao, China; 5 School of Biological Sciences, University of East Anglia, Norwich Research Park 6106, Norwich, United Kingdom; 6 Inspur Smart Supply Chain Technology (Shandong) Co, LTD, Jinan, China; Connecticut Agricultural Experiment Station, New Haven, Connecticut, USA

**Keywords:** *S. thunbergii*, epiphytic bacteria, dehydration stress, microbial ecology

## Abstract

Intertidal macroalgae and their epiphytic bacteria experience periodic dehydration-rehydration cycles due to tidal fluctuations. The influence of tidal dehydration on algal epiphytic bacteria remains poorly understood. This study investigated the effect of tidal dehydration on epiphytic bacterial communities of macroalga *Sargassum thunbergii*. While tidal dehydration had a small impact on the composition of the epiphytic bacterial community of *S. thunbergii*, it significantly influenced community diversity, abundance of dominant taxa, and some predicted functional genes. Specifically, the abundance of Proteobacteria and *Granulosicoccus* increased markedly, whereas that of Cyanobacteria, *Litoreibacter,* and Sva0996_marine_group decreased significantly. The abundance of *Marinomonas* exhibited a trend of initial decrease, followed by subsequent increase. Predictive functional analysis suggested that the bacterial community adapted to dehydration stress by regulating genes involved in energy, nitrogen, and sulfur metabolism. The shifts in the bacterial community following dehydration stress may result from the inherent differential stress tolerance among bacterial taxa and host-mediated facilitation through algal metabolic adjustments that selectively favored specific groups. This study revealed the structural and functional response of the epiphytic bacterial community of macroalgae in intertidal zones to dehydration stress.

**IMPORTANCE:**

The adaptive mechanisms of the intertidal macroalgal-epiphytic bacterial symbiotic system to periodic tidal dehydration stress play a crucial role in maintaining coastal ecosystem stability. Although numerous studies have investigated the effects of tidal dehydration on intertidal macroalgae, the impact of dehydration on the epiphytic bacteria has received much less attention. Our investigation revealed that tidal dehydration stress significantly alters both the community structure and metabolic functions of the epiphytic bacteria on *Sargassum thunbergii*. Notably, dehydration stress selectively enriched stress-tolerant bacterial taxa and induced metabolic reprogramming, particularly in energy, nitrogen, and sulfur cycling pathways. These microbial responses demonstrate not only bacterial stress adaptation strategies but also suggest potential host-mediated regulation within the algal-bacterial symbiotic system. These findings provide novel insights into the ecological adaptability mechanisms of intertidal ecosystems under environmental stress.

## INTRODUCTION

Macroalgae are key components of marine ecosystems, supporting marine biodiversity and global biogeochemical cycles (1, 2). The epiphytic bacterial communities colonizing macroalgal surfaces establish complex symbiotic relationships with their hosts (3, 4). In intertidal zones, periodic tidal fluctuations lead to dehydration stress, significantly impacting both macroalgae and their epiphytic bacteria. Understanding these effects is crucial for elucidating adaptation mechanisms of coastal ecosystems in this unique environment.

Intertidal macroalgae exhibit multi-level adaptations to dehydration stress. Physiologically, *Porphyra haitanensis* can avoid light damage by quickly closing the photosynthetic system, activating S-adenosyl-L-methionine (SAM)-dependent methyltransferase coupled antioxidant system to remove active oxygen, and maintaining ribosome biosynthesis activity to achieve rapid repair after rehydration, thus forming a systematic adaptive strategy (5). Molecular adaptations involve regulating photosynthetic and antioxidant-related proteins (e.g., aldolase I, chaperone proteins) and their associated genes to adapt to dehydration stress (6, 7). Notably, high-intertidal species (e.g., *Pyropia yezoensis*) exhibit greater dehydration tolerance compared to low-intertidal species (e.g., *Sargassum fusiforme*) (8).

Environmental factors can directly or indirectly affect epiphytic microbiota of marine algae. Temperature shifts can alter the microenvironment, promoting the proliferation of pathogenic bacteria (9), while salinity fluctuations can restructure the dominant bacterial populations of kelp (10, 11). UV-B radiation enhances environmental adaptability and resistance of the epiphytic bacterial communities of *S. thunbergii* (12).

However, current understanding of the effects of dehydration stress on epiphytic bacteria is mainly derived from terrestrial plant systems. Drought affects the rhizosphere bacterial communities directly through soil physicochemical changes and indirectly through root exudates. Under drought stress, Proteobacteria and Firmicutes are significantly enriched in the rhizosphere soil, which can improve drought resistance by producing growth regulators, enhancing mineral solubility, or forming thicker peptidoglycan cell walls (13, 14). In addition, *Pseudomonas* can promote the growth of tomato and change its root exudates, which can significantly induce the growth, group swimming, and biofilm formation of the bacteria, indicating that host metabolism also regulates the structure of bacterial community (15).

Drought functionally enriches bacterial genes for lipid metabolism, signal transduction, and defense mechanisms, which are critical adaptations for stress response (16, 17). Additionally, moderate drought can increase the diversity of rhizosphere bacteria and the relative abundance of specific bacteria, while severe drought can reduce the resilience of the plant root bacterial community (18–21). Remarkably, bacterial communities exhibit “stress memory,” with drought-adapted populations showing faster functional recovery after rehydration (22, 23). Furthermore, the impact of drought on epiphytic bacterial communities varies across host species. In temperate and tropical grasses, phyllosphere bacterial diversity declines, with an increase in the abundance of potential pathogenic bacteria, which may be related to the microenvironmental changes caused by stomatal closure under drought conditions (24).

Despite significant progress in terrestrial plants, there is still a lack of research on the responses of epiphytic bacteria of intertidal macroalgae to dehydration stress. We hypothesize that tidal dehydration stress would alter both the structure and functioning of the epiphytic bacterial community on marine algae. *S. thunbergii*, a dominant brown macroalga in China’s intertidal zones (25), represents an ecologically and economically important species (26). This study investigated the effect of tidal dehydration on the structure and function of epiphytic bacteria on *S. thunbergii*. Our findings will contribute to elucidating the fundamental mechanisms of macroalgae-bacteria adaptation to environmental stress and provide scientific support for intertidal ecosystem conservation.

## RESULTS

**Fig 1:**
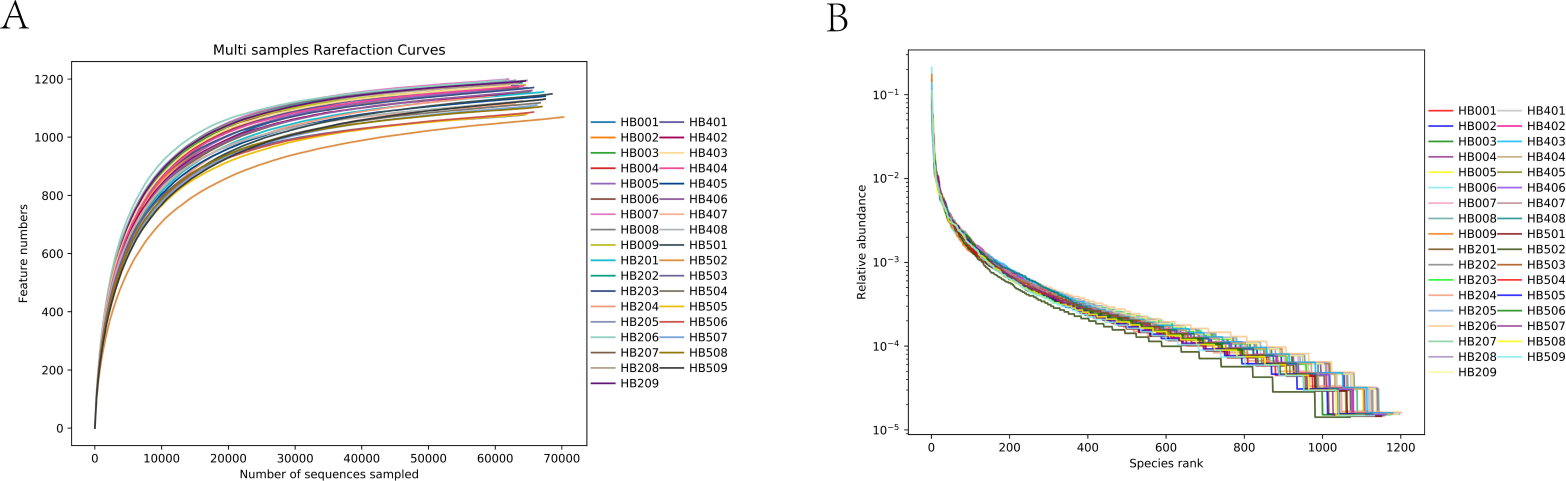
Rarefaction curve and rank abundance curve analysis of all epiphytic bacterial samples on *S. thunbergii* under different tidal dehydration conditions. (**A**) Rarefaction curves. (**B**) Rank abundance curves (HB001–HB009: samples of 0 h dehydration; HB201–HB209: samples of 2 h dehydration; HB401–HB408: samples of 4 h dehydration; HB501–HB509: samples of 1 h rehydration).

A total of 2,800,456 pairs of reads were obtained from all 35 samples. After removing low-quality and mismatched sequences, an average of 79,808 clean reads (range: 79,443–802,13) were retained per sample, and finally, an average of 75,238 effective reads (range: 72,415–76,394) per sample (Table 1) were obtained after removing chimera sequences, such as chloroplasts and mitochondria. All samples were clustered at a 97% similarity threshold, yielding a total of 1,282 OTUs. The dilution and rank abundance curves indicated that the sequencing depth was sufficient for effective bacterial community characterization (Fig. 1).

**TABLE 1:** Number of reads obtained from the sequencing of epiphytic bacterial samples on *S. thunbergii* under different tidal dehydration conditions^*a*^

Samples	Raw reads				Clean reads				Effective reads			
	HB0	HB2	HB4	HB5	HB0	HB2	HB4	HB5	HB0	HB2	HB4	HB5
Replicate 1	80,101	80,005	79,851	80,427	79,895	79,797	79,632	80,213	74,919	76,374	74,712	76,070
Replicate 2	79,751	80,213	80,144	79,713	79,538	80,018	79,932	79,515	74,555	75,577	74,241	76,025
Replicate 3	80,149	80,175	79,795	79,956	79,931	79,947	79,586	79,748	75,353	76,195	74,201	74,208
Replicate 4	80,069	80,034	79,658	80,146	79,855	79,843	79,455	79,962	75,382	72,415	75,191	75,447
Replicate 5	80,200	80,037	79,954	79,723	80,019	79,815	79,786	79,530	75,686	75,139	75,550	74,602
Replicate 6	79,875	79,648	79,991	79,976	79,638	79,443	79,773	79,803	76,394	75,375	75,272	75,093
Replicate 7	80,130	80,328	79,815	80,321	79,914	80,124	79,628	80,145	75,102	76,281	73,479	75,223
Replicate 8	80,119	79,861	80,157	80,217	79,907	79,638	79,960	80,033	75,904	75,145	74,239	75,520
Replicate 9	79,995	79,836	–	80,086	79,777	79,596	–	79,870	76,242	76,173	–	76,045

^*a*^ Due to failed DNA quality control, only eight replicates are available for the HB4 group. “–” denotes one sample omitted because of inadequate DNA quality.

### Bacterial community diversity

**Fig 2:**
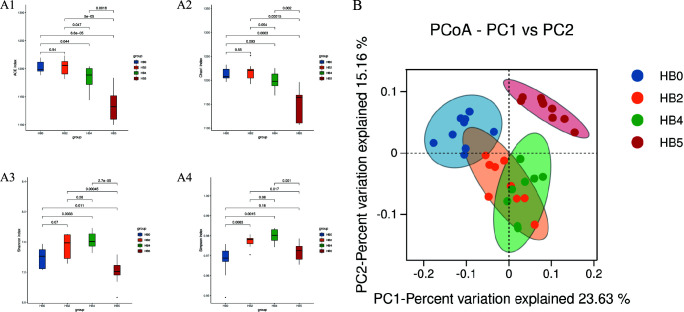
The α diversity indices and β-diversity of epiphytic bacteria of *S. thunbergii* under different tidal dehydration conditions. (**A**) α-Diversity (Student’s *t*-test, *P* < 0.05) (**A1**: Ace; **A2**: Chao 1; **A3**: Shannon; **A4**: Simpson); (**B**): β-diversity (principal coordinates analysis [PCoA] result based on Bray-Curtis distances, permutational multivariate analysis of variance [PERMANOVA], *P* < 0.05).

The analysis of α-diversity for epiphytic bacterial samples on *S. thunbergii* under different dehydration conditions (Fig. 2A and Table 2) indicated that the ACE and Chao1 indices showed no significant changes during the first 0–2 h of dehydration. However, a significant decrease was observed from 2 to 1 h after rehydration (Student’s *t*-test, *P* < 0.05), indicating a continuous decline in species richness of the epiphytic bacterial community on *S. thunbergii* that did not recover after rehydration. In contrast, the Shannon and Simpson indices increased significantly during the dehydration period but decreased after rehydration (*P* < 0.05), suggesting that the bacterial diversity and richness respond differentially at different time points during the dehydration-rehydration process.

**TABLE 2:** Indices of ACE, Chao1, Simpson, and Shannon of epiphytic bacteria of *S. thunbergii* under different dehydration conditions

Group	Ace	Chao1	Simpson	Shannon
HB0	1202.6563	1212.7580	7.3989	0.9674
HB2	1203.1460	1217.1136	7.5356	0.9770
HB4	1182.7642	1198.8364	7.6177	0.9800
HB5	1135.2544	1149.0286	7.1972	0.9721

Additionally, the β-diversity based PCoA analysis and Adonis/Anosim tests (Fig. 2B) revealed a distinct clustering of epiphytic bacterial communities of *S. thunbergii*, with significant differences observed between groups (*P* < 0.05). Water loss stress accounted for 38.1% of the variance in these epiphytic bacterial communities, indicating that water stress had a significant impact on the structure of epiphytic bacterial communities of *S. thunbergii*.

### Bacterial community composition

**Fig 3:**
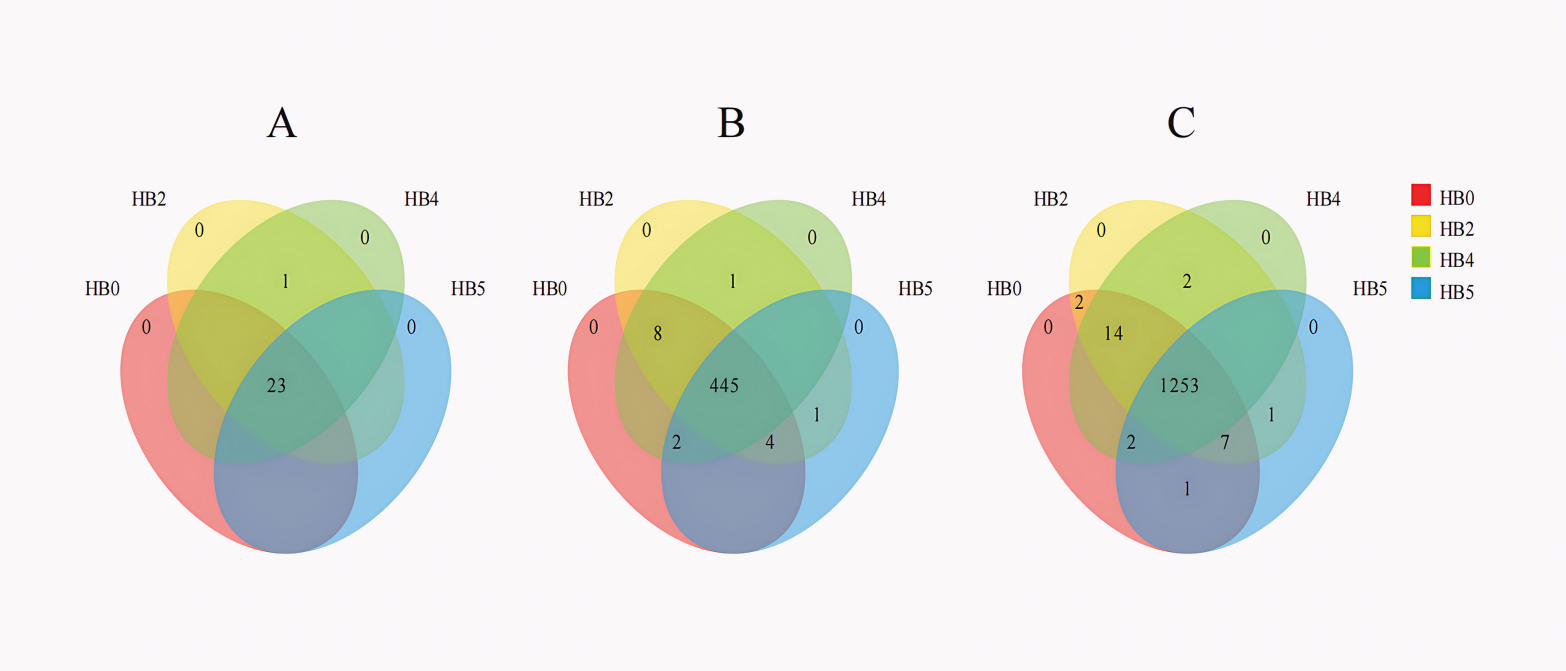
The variations in the shared and specific epiphytic bacteria of *S. thunbergii* under different tidal dehydration conditions. (**A**) Phylum level, (**B**) genus level, and (**C**) OTU level.

High-throughput sequencing revealed 24 phyla and 461 genera in the epiphytic bacterial communities of *S. thunbergii*. Notably, 23 phyla (95.8% of total) and 445 genera (96.5% of total) were shared across all experimental groups. Only Deinococcota is unique to the H2 and H4 groups (Fig. 3A), while only 16 genera (3.5% of total), including *Flavobacterium*, *Asaia*, and *Thermu,* showed intergroup variations (Fig. 3B). The core microbiome was particularly stable, with 97.7% of OTUs (1,253) being shared among all groups (Fig. 3C). This suggests that dehydration stress has minimal impact on the composition of the epiphytic bacterial communities of *S. thunbergii* at various taxonomic levels.

**Fig 4:**
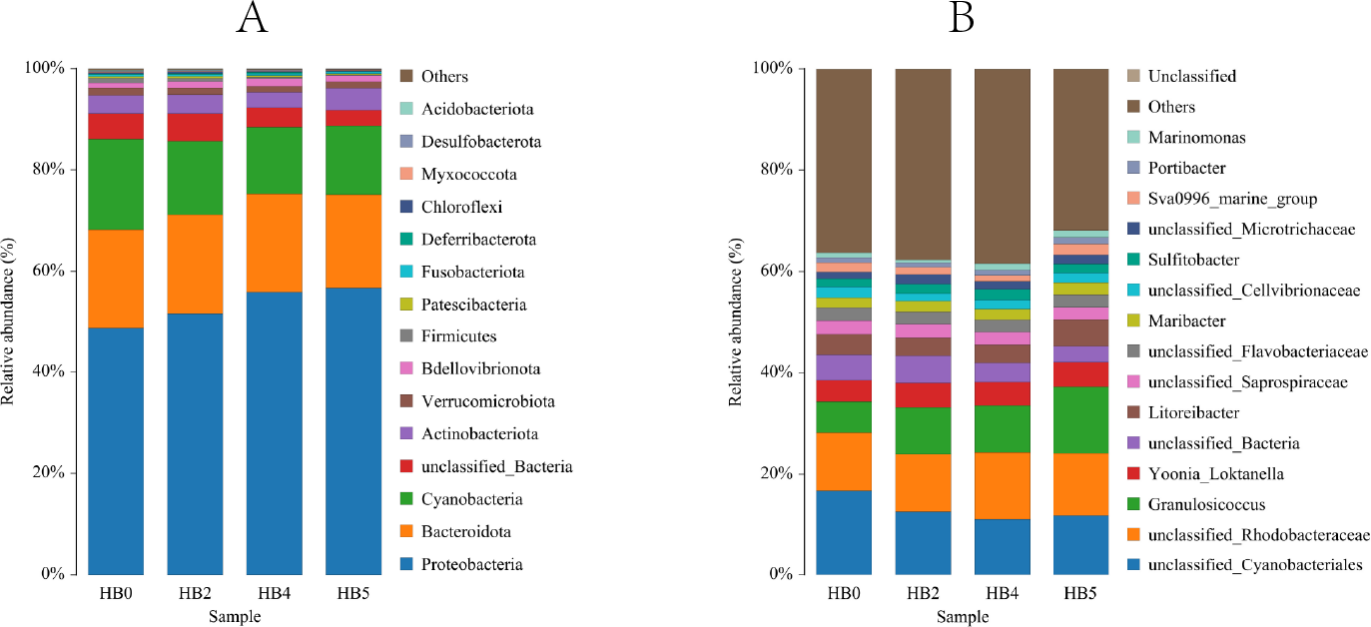
The variations in the relative abundance of the top 15 epiphytic bacterial taxa of *S. thunbergii* under different tidal dehydration conditions. (**A**) Phylum level, (**B**) genus level (The remaining species are grouped as “others,” Wilcoxon rank-sum test, *P* < 0.05).

The results based on the Wilcoxon rank-sum test presented in Fig. 4 illustrated the top 15 dominant taxa of epiphytic bacteria on *S. thunbergii* at the phylum and genus levels. The findings indicated that while the species composition of the epiphytic bacterial communities of *S. thunbergii* remained largely consistent across different dehydration conditions, significant variations in the relative abundances of certain dominant taxa were observed (*P* < 0.05).

At the phylum level, Proteobacteria emerged as the most dominant taxa, accounting for 48.66% to 56.73% of total abundance, followed by Bacteroidota (18.40% to 19.31%) and Cyanobacteria (13.17% to 17.88%). Other phyla with relative abundances exceeding 1% included Actinobacteria, Verrucomicrobiota, and Bdellovibrionota.

At the genus level, *Granulosicoccus* was the most dominant across all experimental groups (6.13% to 13.10%), followed by *Yoonia_Loktanella* (4.22% to 4.86%) and *Litoreibacter* (3.52% to 5.33%). Other genera with relative abundances greater than 1% included *Maribacter*, *Sulfitobacter*, *Sva0996_marine_group*, *Portibacter*, and *Marinomonas*.

Further statistical analysis (Wilcoxon rank-sum test) revealed that dehydration stress significantly affected the relative abundances of five phyla and four genera among the top 15 dominant taxa of epiphytic bacteria on *S. thunbergii* (*P <* 0.05). Specifically, at the phylum level, as dehydration time prolonged, the relative abundances of Proteobacteria and Bacteroidota significantly increased (*P <* 0.05), while those of Cyanobacteria, Firmicutes, and Acidobacteria significantly decreased (*P <* 0.05). At the genus level, the relative abundance of Sva0996_marine_group showed a significant decrease with increasing dehydration duration but markedly recovered to pre-stress levels after rehydration (*P <* 0.05). The relative abundance of *Granulosicoccus* remained relatively stable at both 2 and 4 h of dehydration, which were higher than those at 0 h and further increased significantly upon rehydration (*P <* 0.05). *Marinomonas* reached its lowest relative abundance at 2 h of dehydration but increased significantly thereafter (*P <* 0.05). Meanwhile, *Litoreibacter*’s relative abundance displayed reduced abundance at 2 h and 4 h of dehydration compared to the initial state (0 h) but recovered significantly after rehydration (*P <* 0.05).

### Indicator species

**Fig 5:**
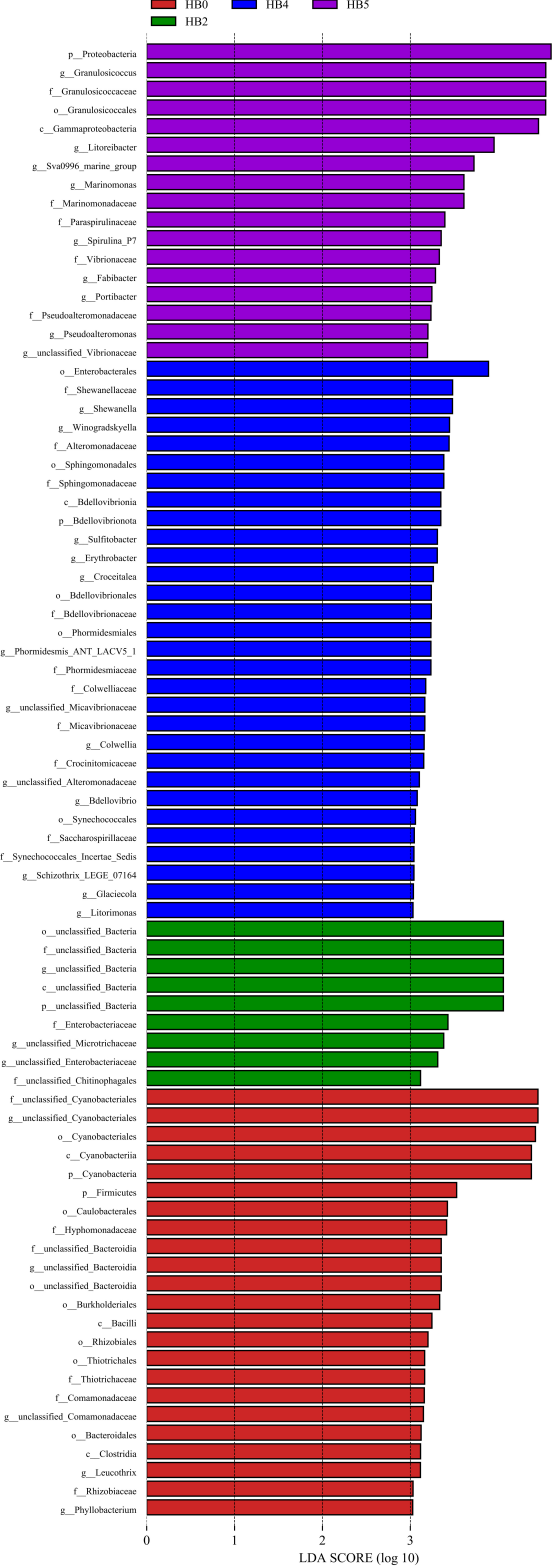
The variations in the indicator species of epiphytic bacteria on *S. thunbergii* under different tidal dehydration conditions (The colors of the bars represent the groups, and the lengths of the bars represent the contributions of the indicator species, LEfSe analysis, Kruskal-Wallis test (*P* < 0.05), Wilcoxon test (*P* < 0.05), LDA > 3.0).

Linear discriminant analysis effect size (LEfSe) analysis (Kruskal-Wallis rank-sum test, Wilcoxon rank-sum test, and line discriminant analysis) showed that there were significant differences in the indicator species (LDA > 3.0, *P* < 0.05) of the epiphytic bacteria on *S. thunbergii* under different dehydration conditions (Fig. 5). It was found that the 4 h of dehydration group had the highest number of biomarkers (29), followed by the 0 h group (22) and the 1 h of rehydration group (17), while the 2 h of dehydration group had the lowest number (8).

Among them, Cyanobacteria and Firmicutes were significantly enriched at 0 h of dehydration; Bdellovibrionota and Proteobacteria showed higher abundance after losing water for 4 h of dehydration and 1 h of rehydration, respectively.

At the genus level, *Leucothrix* and *Phyllobacterium* were abundant at 0 h of dehydration. At 2 h of dehydration, there was no significantly enriched bacteria. At 4 h of dehydration, the main enriched epiphytic bacteria included *Shewanella*, *Winogradskyella,* and *Sulfitobacter*, etc., while the main enriched epiphytic bacteria during 1 h of rehydration were *Granulosicoccus*, *Litoreibacter*, *Sva0996_marine_group, Marinomonas*, etc. The results indicated significant differences in the enriched epiphytic bacterial taxa of the *S. thunbergii* under different dehydration conditions (*P<* 0.05).

### Functional prediction analysis

**Fig 6:**
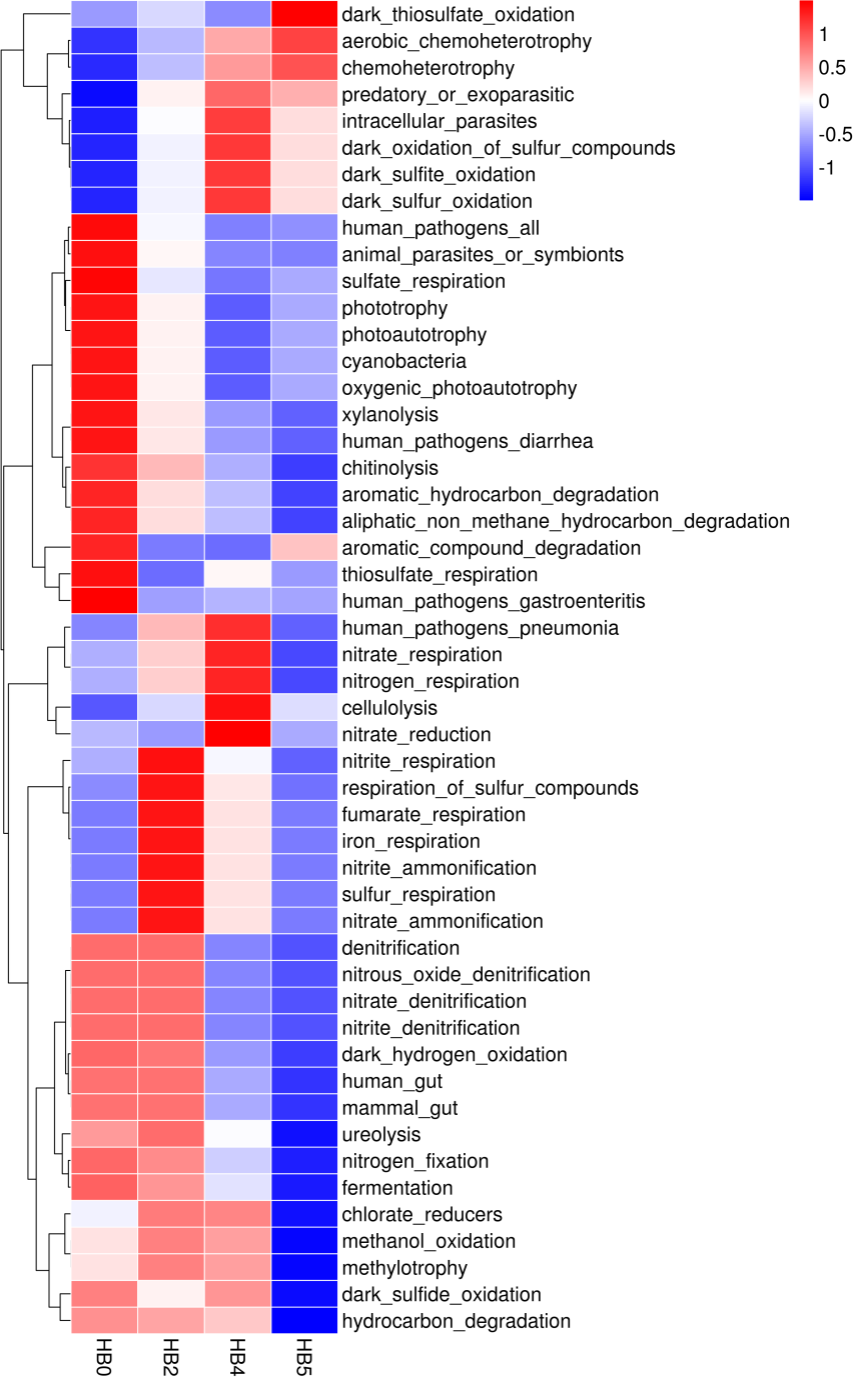
The heatmap of the variations in abundances of predicted functional genes of epiphytic bacteria on *S. thunbergii* under different tidal dehydration conditions predicted by FAPROTAX (Kruskal-Wallis rank-sum test, *P* < 0.05) based on the SILVA database (The vertical axis indicates the sample groups at different dehydration times. The horizontal axis indicates each functional group of the elemental cycle. Red and blue indicate the functional abundance; the larger the value, the higher the predicted functional abundance).

Functional annotation of prokaryotic taxa (FAPROTAX)analysis (Kruskal-Wallis rank-sum test) revealed significant changes in the abundance of some predicted functional genes of the epiphytic bacterial community on *S. thunbergii* during tidal dehydration and rehydration (*P <* 0.05) (Fig. 6). Among the 50 detected functional genes, the abundance of most genes, such as Nitrate-dentification, Nitrite_dentification, nitrogen fixation, and cyanobacteria, declined continuously, while the abundance of dark-sulfur-oxidation and dark-sulfite-oxidation continued to increase. The abundance of genes, such as nitrite_spiration, nitrite-ammoniation, and sulfur respiration continued to increase during dehydration but recovered after rehydration.

**Fig 7:**
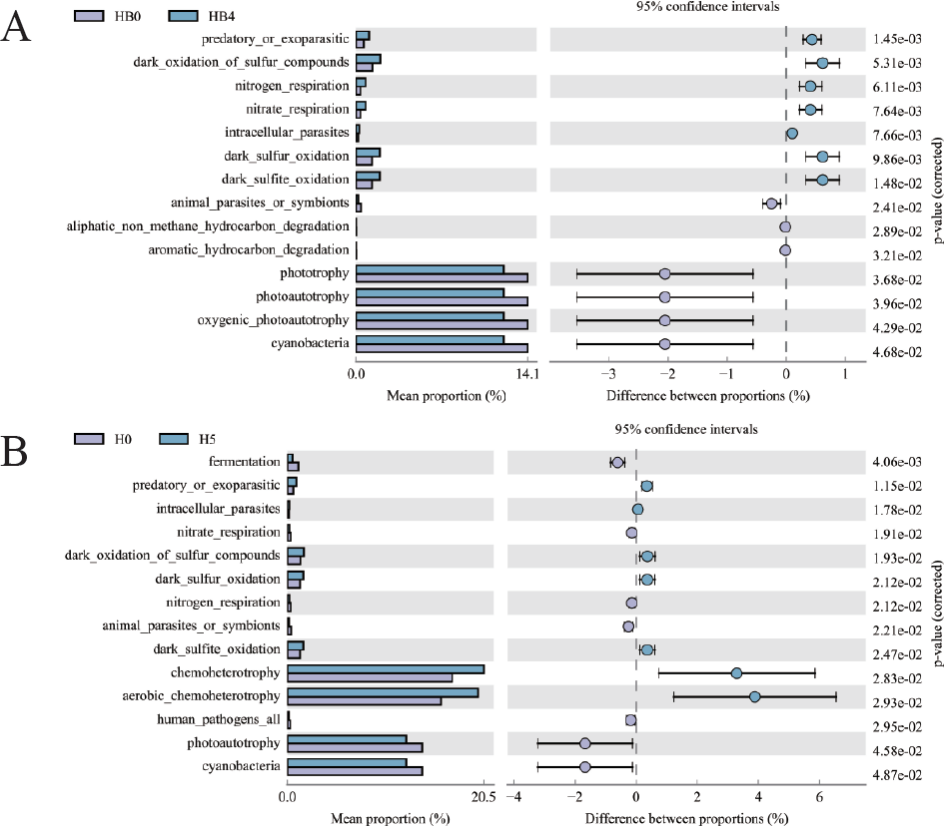
Abundance of predicted functional genes of epiphytic bacteria on *S. thunbergii* with significant changes after 4 h of dehydration (**A**) and after 1 h of rehydration (**B**), compared to 0 h of dehydration (Wilcoxon rank-sum test, *P* < 0.05).

Further statistical analysis (Wilcoxon rank-sum test) indicated no significant changes in the abundance of predicted functional genes of the epiphytic bacterial community of *S. thunbergii* after 2 h of dehydration compared with 0 h of dehydration (*P* < 0.05). However, at 4 h of dehydration, genes related to predation or exoparasitic, nitrogen respiration, nitrate respiration, intracellular parasites, and dark sulfur oxidation pathways were significantly upregulated (*P* < 0.05), while those involved in photoautotrophy, aliphatic non-methane hydrocarbon degradation, cyanobacteria, oxygenic photoautotrophy, and phototrophy were significantly downregulated (*P* < 0.05) (Fig. 7A).

Following 1 h of rehydration, the epiphytic bacterial community of *S. thunbergii* showed elevated gene abundances for chemoheterotrophy, aerobic chemoheterotrophy, and dark sulfur metabolic pathways compared with 0 h of dehydration (*P* < 0.05). However, functions, such as photoautotrophy and cyanobacteria, showed a decline (Fig. 7B). These results suggest even after rehydration, the functional abundance of epiphytic bacteria on *S. thunbergii* could not fully recover to levels observed before dehydration.

## DISCUSSION

### Effect of tidal dehydration on the community structure of the epiphytic bacterial communities on *S. thunbergii*

This study revealed that tidal dehydration had limited impact on the composition of epiphytic bacterial communities of the intertidal macroalga *S. thunbergii*. While the core bacterial assemblage remained stable, the diversity and the abundance distribution among dominant taxa was altered significantly. Specifically, short-term dehydration (0–2 h) did not significantly alter epiphytic bacterial richness on *S. thunbergii*, suggesting most bacterial species tolerated initial dehydration. However, the continued decline after 2 h reflected the elimination of dehydration-sensitive species and a gradual dominance by drought-resistant taxa. Interestingly, after 4 h of dehydration, diversity increased despite reduced richness, likely due to that dehydration stress reduces the competitive dominance of previously abundant bacterial taxa, leading to increased community evenness that outweighs the loss of rare species. Additionally, dehydration may also stimulate *S. thunbergii* to release diverse metabolites, further promoting bacterial diversity. It is worth mentioning that although the epiphytic bacterial diversity of *S. thunbergii* largely recovered after rehydration, richness remained significantly lower than initial levels. This indicates that although the bacterial community exhibits a certain degree of stability under dehydration stress, complete recovery is a time-consuming process, with some dehydration-sensitive taxa recovering gradually over an extended period. However, due to the constraints of the sampling timeline in this study, it remains unclear whether the observed decline in the richness of the algal epiphytic bacterial community after 1 h rehydration represents a permanent loss or a transitional state capable of gradual recovery over an extended period. This will be specifically investigated in future studies through longer-term dehydration-rehydration experiments. Furthermore, it should be noted that the Shannon index of the epiphytic bacterial community in this study exhibited a fluctuation pattern of “first decline and then recovery,” which reflects not merely the simple loss and regain of community diversity; it indicates a shift in the driving factors of the bacterial community. During the dehydration stage, environmental filtering eliminated sensitive groups, leading to a simultaneous decline in richness and evenness. After rehydration, as species richness did not fully recover, the recovery of the Shannon index was mainly due to an increase in evenness. This suggests that the community did not return to its original state but rather was reorganized through random dispersion and competition (27, 28) to form a new community with a different composition but similar evenness. Therefore, the recovery of the Shannon index cannot be equated with the complete restoration of community structure or function, which implies that the assessment of microbial community resilience requires the integration of multiple indicators, such as composition and function.

Water loss stress also leads to changes in the abundance of dominant taxa in the epiphytic bacterial community of *S. thunbergii.* The core epiphytic bacterial community of *S. thunbergii*, Proteobacteria, Bacteroidota, and Cyanobacteria, which are typical marine epiphytic bacterial taxa (4), exhibited distinct responses to dehydration stress. The marked increase in *Proteobacteria* abundance under dehydration conditions is consistent with previous findings on the enrichment of *Proteobacteria* in drought-stressed rhizosphere bacterial communities (29, 30). This may be because Proteobacteria can alleviate the impact of dehydration by producing growth regulators and forming a thicker peptidoglycan cell wall (13, 14), and can also enhance drought tolerance by forming spores (18). In contrast, cyanobacteria, whose photosynthetic products typically support algal growth and microbial biofilm formation, exhibited a significant decline in abundance after dehydration, likely due to its high sensitivity to dehydration. Dehydration stress also induced notable shifts in dominant bacterial genera. The abundance of *Granulosicoccus* increased significantly under dehydration conditions, likely because as an obligate aerobe (31, 32), it possesses greater competitive and stress-tolerant advantages over bacteria adapted to the lower and fluctuating dissolved oxygen levels in sea water. This advantage becomes particularly pronounced during low-tide exposure to the atmospheric oxygen in the intertidal zone, thereby facilitating its proliferation. Conversely, the abundance of *Litoreibacter* (which produces sulfur/nitrogen compounds (33) and Sva0996_marine_group (involved in organic nitrogen cycling and the uptake of phytoplankton-derived dissolved proteins (34) decreased, possibly due to dehydration-induced metabolic inhibition. Moreover, the abundance of *Marinomonas* exhibited an initial decline followed by recovery, possibly owing to its metabolic versatility (e.g., synthesizing osmoprotectants) and adaptability, enabling gradual acclimation to stress (35).

These findings demonstrate that dehydration stress directly alters the epiphytic bacterial community on *S. thunbergii* through physical screening, resulting in the decline of stress-sensitive taxa (e.g., Cyanobacteria) and the enrichment of tolerant bacterial taxa (e.g., Proteobacteria and *Granulosicoccus*). Additionally, host-mediated mechanisms, such as algal metabolite production, may further facilitate the proliferation of specific bacterial taxa, enhancing their ability to assist in stress resistance.

### Response of function in the epiphytic bacterial community of *S. thunbergii* to dehydration stress

This study revealed significant changes in the abundance of some predicted functional genes of the epiphytic bacterial community on *S. thunbergii* under tidal dehydration stress. First, the abundance of carbon fixation genes (photoautotrophy and oxygenic photoautotrophy) significantly decreased, suggesting a drought-induced energy conservation strategy (36). Similarly, the abundance of hydrocarbon degradation genes (including aliphatic non-methane and aromatic hydrocarbon degradation) also declined significantly, consistent with previous findings that extreme drought reduces the abundance of functional groups involved in aromatic compound and lignin degradation in soil (37).

It is worth noting that these functional changes corresponded with taxonomic shifts of epiphytic bacteria of *S. thunbergii* under dehydration stress. For instance, dehydration not only significantly reduced the relative abundance of phototrophic bacteria, such as Cyanobacteria, but also heterotrophic bacteria like Firmicutes and Acidobacteria. These taxa normally provide essential nutrients (CO_2_, fixed nitrogen) and primary production for host algae (38, 39), suggesting potential metabolic impacts on the algal-bacterial symbiont.

Second, the abundance of sulfur and nitrogen metabolism genes of the epiphytic bacteria of *S. thunbergii* changed significantly during the dehydration process. The abundance of sulfur oxidation genes (dark_sulfur_oxidation, dark_sulfite_oxidation) continuously increased during the dehydration period. Previous studies demonstrate drought-stressed plants (e.g., sweet pepper) undergo adaptive modifications in glutathione and sulfur metabolic pathways (40). Moreover, drought stress induces a significant accumulation of reactive oxygen species (ROS) in plant and bacteria (41). Consequently, the upregulation of bacterial sulfur metabolism genes observed in this study may represent a cooperative response mechanism aimed at alleviating ROS-induced cellular damage in the host algae under drought conditions. Notably, this study observed a significant enrichment of the indicator taxa *Shewanella* and *Sulfitobacter* under dehydration conditions. Interestingly, although *Shewanella* is commonly recognized as a sulfate-reducing bacterium, it preferentially oxidizes thiosulfate over performing reduction in the presence of oxygen (42). In contrast, *Sulfitobacter*, as a classic sulfur-oxidizing bacterium, is capable of oxidizing thiosulfate, sulfur, and sulfite to sulfate (43). It is widely recognized that sulfur-containing compounds are closely associated with biological antioxidant mechanisms (44). Therefore, the observed changes in these indicator taxa may be linked to the response of the antioxidant system under dehydration stress. This interpretation further supports the enhanced sulfur oxidation function predicted in our study.

Conversely, the abundance of the predicted functional genes involved in nitrate denitrification, nitrite denitrification, and nitrogen fixation continuously decreased during dehydration. This reduction is likely attributed to the oxygen sensitivity of nitrifying bacteria and nitrogenase, which is only active under strictly anaerobic conditions. The exposure to oxygen during tidal dehydration might have led to the decline of these functions. Similar trends have been reported in drought-tolerant rice mutants, which exhibit heightened nitrogen sensitivity and reduced nitrogen use efficiency under stress (45). Interestingly, the upregulation of nitrite respiration and nitrite ammonification genes suggests a potential metabolic coordination between epiphytic bacteria and the host alga to compensate for nitrogen metabolism disruptions in response to drought stress. This is supported by studies demonstrating that the drought-responsive transcription factor, drought and salt tolerance (DST), enhances nitrate assimilation in rice by activating nitrate reductase genes, thereby improving stress tolerance (45). In summary, the shifts in the abundance of some predicted functional genes within the epiphytic bacteria of *S. thunbergii* may be due to their own responses to dehydration and the cooperative responses to the metabolic changes of the host algae under drought stress, which helps to enhance the overall resistance of the host algae-epiphytic bacteria symbiont to environmental stress.

Additionally, numerous studies have shown that intertidal macroalgae exhibit differential protein expression and metabolite abundance during natural tide-induced cyclic dehydration and rehydration. These changes are primarily implicated in antioxidant systems and osmoregulatory mechanisms, which are recognized as key strategies for resisting desiccation stress (46–48). Recent research has revealed that intertidal algae may enhance their environmental adaptability through genetic exchange with symbiotic microorganisms. For example, the symbiotic bacterium *Saccharothrix* sp., isolated from *Neoporphyra haitanensis*, has been shown to significantly improve the heat tolerance of the alga by regulating host genes involved in proline synthesis, redox homeostasis, and protein folding^49^—processes that are also closely associated with the mechanisms by which intertidal macroalgae resist dehydration stress. Furthermore, Chen et al. found that the ancestor of *N. haitanensis* acquired a unique lipoxygenase gene family involved in complex chemical defense, as well as a large number of stress tolerance-related genes, from epiphytic bacteria (50). It is worth noting that proline, a nitrogen-containing compound, is closely linked to nitrogen cycle metabolism, while sulfur is involved in the synthesis of various antioxidants. In this study, we observed significant changes in predicted nitrogen- and sulfur-related functional pathways in the epiphytic bacterial community of *S. thunbergii*, suggesting that this may represent a coordinated adaptation strategy of the macroalgae–epiphytic bacteria symbiont to the intertidal environment.

This study reveals the effect of tidal dehydration on epiphytic bacterial communities of *S. thunbergii*. While the overall community structure demonstrates relative stability under different water loss conditions, significant fluctuations in both dominant taxa abundance and predicted functional gene expression were observed. These shifts may reflect not only the intrinsic sensitivity of epiphytic bacteria to dehydration but also their coordinated metabolic adaptations with the host alga during stress conditions. Our findings provide novel insights into the adaptive mechanisms of algal-bacterial symbiotic systems in intertidal environments.

However, this study has several limitations. First, the experimental design included only a single rehydration time point, which prevents a full resolution of bacterial community dynamics across the dehydration-rehydration cycle. Second, the functional analysis based on FAPROTAX relies on taxonomic identification from 16S rRNA gene sequences to infer function, rather than direct measurement of functional genes, and the database is inherently biased toward terrestrial and soil bacteria, thereby limiting its accuracy for marine bacterial functions. Moreover, host-mediated regulation, algal metabolite release, or coordinated nitrogen metabolism lacks direct experimental support. To address these issues, future work will involve establishing a laboratory-based simulated tidal system, collecting samples at multiple time points, and combining metabolomic, metagenomic, or metatranscriptomic and physiological assays. This integrated approach will allow simultaneous analysis of metabolic interactions between the host macroalgae and its associated bacterial community, thereby helping to accurately elucidate the metabolic responses and underlying mechanisms of the intertidal macroalgae–epiphytic bacterium symbiont under dehydration stress.

## MATERIALS AND METHODS

### Sample collection and processing

**Fig 8:**
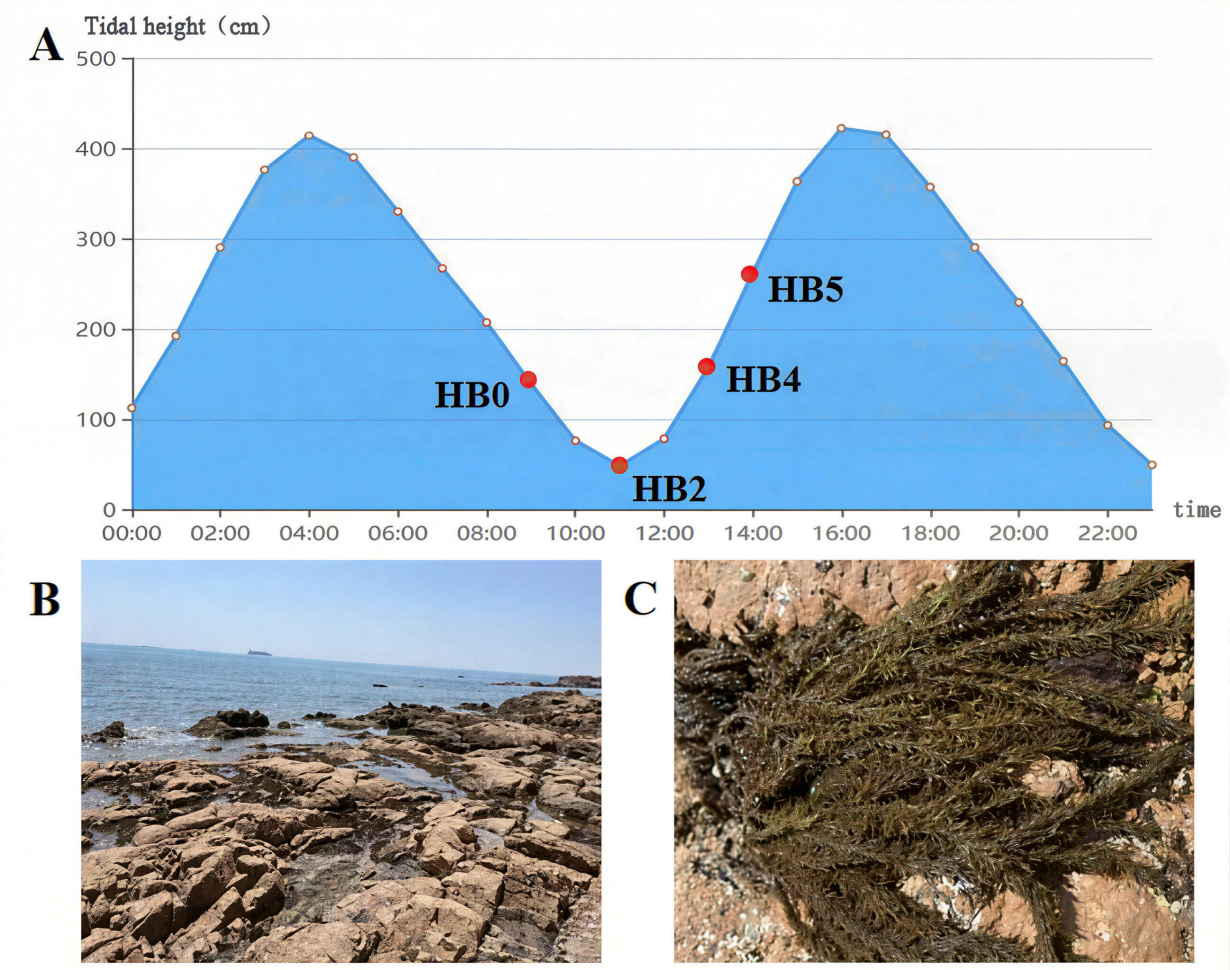
Sampling information: (**A**) Sampling time (**B**) Intertidal environment of sampling site. (**C**) *S. thunbergii.*

Healthy *S. thunbergii* samples (approximately 25 g each) were randomly collected from a 100 m × 10 m rectangular area in the intertidal zone of Taiping Cape (120°35′ E, 36°05′ N), Qingdao, on 16 April 2022. Sampling was conducted at four time points: 0 h (initial exposure), 2 h, 4 h of dehydration, and 1 h after rehydration (Fig. 8). Algal samples were immediately placed in sterile bags, stored on ice in a portable container, and transported to the laboratory.

Each sample was rinsed with sterile seawater, and a 25 g sample was weighed and shaken with 70 mL of PBS buffer (0.01 M, pH 7.4) for 30 min (200 r/min). The resulting suspension was sequentially filtered through 500-mesh bolting cloth and 0.22-μm membranes to collect bacteria, which were stored at −80 °C for further analysis (51). Samples were labeled as follows: HB0 (0 h control), HB2 (dehydration for 2 h), HB4 (dehydration for 4 h), and HB5 (rehydration for 1 h). Each treatment group included nine independent algal individuals.

### DNA sequencing

The DNA sequencing was conducted by Biomarker Technologies Co., LTD (China). DNA was extracted using the TGuide S96 Magnetic Soil and Stool DNA Kit (Tiangen Biotech, Beijing), and DNA quality was assessed using a NanoDrop 2000 spectrophotometer (Thermo Fisher Scientific, USA). The V3–V4 region of the 16S rRNA gene was amplified using the 338F (5′-ACTCCTACGGGAGGCAGCAG-3′) / 806R (5′-GGACTACHVGGGTWTCTAAT-3′) primers and sequencing was performed on the Illumina Novaseq 6000 platform.

### Data nalysis

Raw sequencing data were processed using FLASH (v1.2.11) for sequence merging and Trimmomatic (version 0.33) for quality control. Chimeric sequences and chloroplast/mitochondrial sequences were removed using UCHIME (version 4.2). USEARCH (version 10.0) was employed to cluster OTUs (Operational Taxonomic Units) at a 97% similarity threshold (52), followed by taxonomic annotation based on the SILVA database (version 128) (threshold: 0.8). The α-diversity indices (Ace, Chao1, Shannon, Simpson) were calculated using QIIME 2 (version 2.0), and intergroup differences in α-diversity were assessed using Student’s *t*-test (*P* < 0.05). β-Diversity was assessed via PCoA and PERMANOVA/analysis of similarities (ANOSIM) tests (*P* < 0.05) were applied to evaluate intergroup differences. Wilcoxon rank-sum test (*P* < 0.05) was used to analyze significant differences in the relative abundance of dominant bacterial taxa at the phylum and genus levels. LEfSe analysis was employed to identify differentially abundant microbial taxa across groups through a hierarchical screening from phylum to genus. Statistical significance was first assessed using the Kruskal-Wallis and Wilcoxon rank-sum tests (*P* < 0.05), followed by linear discriminant analysis (LDA) to evaluate effect sizes (LDA > 3.0). The sequencing data were functionally annotated using the FAPROTAX database.

## Data Availability

The data obtained in this study have been deposited in the National Center for Biotechnology Information (NCBI) (BioProject ID: PRJNA938272).

## References

[1] Krause-Jensen D, Duarte CM. 2016. Substantial role of macroalgae in marine carbon sequestration. Nature Geosci 9:737–742. doi:10.1038/ngeo2790

[2] Pei P, Aslam M, Du H, Liang H, Wang H, Liu X, Chen W. 2021. Environmental factors shape the epiphytic bacterial communities of Gracilariopsis lemaneiformis. Sci Rep 11:8671. doi:10.1038/s41598-021-87977-3 33883606 PMC8060329

[3] Pfister CA, Berlinghof J, Bogan M, Cardini U, Gobet A, Hamon-Giraud P, Hart J, Jimenez N, Siegel A, Stanfield E, Vallet M, Leblanc C, Rousseau C, Thomas F, Stock W, Dittami SM. 2025. Evolutionary history and association with seaweeds shape the genomes and metabolisms of marine bacteria. mSphere 10. doi:10.1128/msphere.00996-24 PMC12188728 40454874

[4] Comba González NB, Niño Corredor AN, López Kleine L, Montoya Castaño D. 2021. Temporal changes of the epiphytic bacteria community from the marine macroalga Ulva lactuca (Santa Marta, Colombian-Caribbean). Curr Microbiol 78:534–543. doi:10.1007/s00284-020-02302-x 33388936

[5] Chen H, Chu J-C, Chen J, Luo Q, Wang H, Lu R, Zhu Z, Yuan G, Yi X, Mao Y, Lu C, Wang Z, Gu D, Jin Z, Zhang C, Weng Z, Li S, Yan X, Yang R. 2022. Insights into the ancient adaptation to intertidal environments by red algae based on a genomic and multiomics investigation of Neoporphyra haitanensis. Mol Biol Evol 39:msab315. doi:10.1093/molbev/msab315 34730826 PMC8752119

[6] López-Cristoffanini C, Zapata J, Gaillard F, Potin P, Correa JA, Contreras-Porcia L. 2015. Identification of proteins involved in dehydration tolerance in the red seaweed Pyropia Orbicularis (Rhodophyta, Bangiales). Proteomics 15:3954–3968. doi:10.1002/pmic.201400625 26154304

[7] Fierro C, López-Cristoffanini C, Meynard A, Lovazzano C, Castañeda F, Guajardo E, Contreras-Porcia L. 2017. Expression profile of desiccation tolerance factors in intertidal seaweed species during the tidal cycle. Planta 245:1149–1164. doi:10.1007/s00425-017-2673-0 28289905

[8] Gao S. 2014. Responses of photosynthesis in intertidal macroalgae to dehydration stress PhD Thesis, University of Chinese Academy of Sciences, Beijing

[9] Stratil SB, Neulinger SC, Knecht H, Friedrichs AK, Wahl M. 2013. Temperature-driven shifts in the epibiotic bacterial community composition of the brown macroalga Fucus vesiculosus. Microbiologyopen 2:338–349. doi:10.1002/mbo3.79 23568841 PMC3633357

[10] Schenk S, Wardrop CG, Parfrey LW. 2025. Abiotic stress destabilizes the bacterial community of sugar kelp, Saccharina latissima (Phaeophyceae). J Phycol 61:840–857. doi:10.1111/jpy.70033 40432552 PMC12351371

[11] Zhang Y, Alam MA, Kong X, Wang Z, Li L, Sun Y, Yuan Z. 2017. Effect of salinity on the microbial community and performance on anaerobic digestion of marine macroalgae. J of Chemical Tech & Biotech 92:2392–2399. doi:10.1002/jctb.5246

[12] Wang J, Yang Z, Lu P, Sun Y, Xue S, Tang X, Xiao H. 2023 Effects of UV-B radiation on epiphytic bacterial communities on male and female Sargassum thunbergii. Sci Rep 13. doi:10.1038/s41598-022-26494-3 PMC9998616 36894683

[13] Liu T, Ye N, Wang X, Das D, Tan Y, You X, Long M, Hu T, Dai L, Zhang J, Chen M. 2021. Drought stress and plant ecotype drive microbiome recruitment in switchgrass rhizosheath. JIPB 63:1753–1774. doi:10.1111/jipb.13154 34288433

[14] Gontia-Mishra I, Sapre S, Kachare S, Tiwari S. 2017. Molecular diversity of 1-aminocyclopropane-1-carboxylate (ACC) deaminase producing PGPR from wheat (Triticum aestivum L.) rhizosphere. Plant Soil 414:213–227. doi:10.1007/s11104-016-3119-3

[15] Zhang H, Zheng D, Hu C, Cheng W, Lei P, Xu H, Gao N. 2023. Certain tomato root exudates induced by Pseudomonas stutzeri NRCB010 enhance its rhizosphere colonization capability. Metabolites 13:664. doi:10.3390/metabo13050664 37233705 PMC10220591

[16] Dai L, Zhang G, Yu Z, Ding H, Xu Y, Zhang Z. 2019. Effect of drought stress and developmental stages on microbial community structure and diversity in peanut rhizosphere soil. Int J Mol Sci 20:2265. doi:10.3390/ijms20092265 31071918 PMC6540327

[17] Wu L, Wang J, Wu H, Chen J, Xiao Z, Qin X, Zhang Z, Lin W. 2018. Comparative metagenomic analysis of rhizosphere microbial community composition and functional potentials under Rehmannia glutinosa consecutive monoculture. Int J Mol Sci 19:2394. doi:10.3390/ijms19082394 30110928 PMC6121535

[18] Xie J, Dawwam GE, Sehim AE, Li X, Wu J, Chen S, Zhang D. 2021. Drought stress triggers shifts in the root microbial community and alters functional categories in the microbial gene pool. Front Microbiol 12. doi:10.3389/fmicb.2021.744897 PMC8566882 34745045

[19] Santos-Medellín C, Liechty Z, Edwards J, Nguyen B, Huang B, Weimer BC, Sundaresan V. 2021. Prolonged drought imparts lasting compositional changes to the rice root microbiome. Nat Plants 7:1065–1077. doi:10.1038/s41477-021-00967-1 34294907

[20] Dao J, Xing Y, Chen C, Chen M, Wang Z. 2023. Adaptation of rhizosphere bacterial communities of drought-resistant sugarcane varieties under different degrees of drought stress. Microbiol Spectr 11. doi:10.1128/spectrum.01184-23 PMC10580969 37698408

[21] Preece C, Verbruggen E, Liu L, Weedon JT, Peñuelas J. 2019. Effects of past and current drought on the composition and diversity of soil microbial communities. Soil Biology and Biochemistry 131:28–39. doi:10.1016/j.soilbio.2018.12.022

[22] Zhou X, Fornara D, Ikenaga M, Akagi I, Zhang R, Jia Z. 2016. The resilience of microbial community under drying and rewetting cycles of three forest soils. Front Microbiol 7:1101. doi:10.3389/fmicb.2016.01101 27486444 PMC4949271

[23] Liu D, Keiblinger KM, Leitner S, Wegner U, Zimmermann M, Fuchs S, Lassek C, Riedel K, Zechmeister-Boltenstern S. 2019. Response of microbial communities and their metabolic functions to drying–rewetting stress in a temperate forest soil. Microorganisms 7:129. doi:10.3390/microorganisms7050129 31086038 PMC6560457

[24] Bechtold EK, Ryan S, Moughan SE, Ranjan R, Nüsslein K. 2021. Phyllosphere community assembly and response to drought stress on common tropical and temperate forage grasses. Appl Environ Microbiol 87. doi:10.1128/AEM.00895-21 PMC8357280 34161142

[25] Zeng C. 2009. Seaweeds in Yellow Sea and Bohai Sea of China. Science Press, Beijing.

[26] Sun Y, Li Q. 2018. Development and utilization of Sargassum thunbergii: a review. Fisheries Science & Technology Information 45:351. doi:10.16446/j.cnki.1001-1994.2018.06.011

[27] Hongyu N, Zhengfeng W, Juyu L, Wanhui Y, Hao S. 2011. New progress in community assembly: community phylogenetic structure combining evolution and ecology. Biodiversity Science. doi:10.3724/SP.J.1003.2011.09275

[28] Zhou J, Chen G-F, Ying K-Z, Jin H, Song J-T, Cai Z-H. 2019. Phycosphere microbial succession patterns and assembly mechanisms in a marine dinoflagellate bloom. Appl Environ Microbiol 85:e00349-19. doi:10.1128/AEM.00349-19 31126952 PMC6643250

[29] Ullah A, Akbar A, Luo Q, Khan AH, Manghwar H, Shaban M, Yang X. 2019. Microbiome diversity in cotton rhizosphere under normal and drought conditions. Microb Ecol 77:429–439. doi:10.1007/s00248-018-1260-7 30196314

[30] Xu L, Naylor D. 2018. Drought delays development of the sorghum root microbiome and enriches for monoderm bacteria. Proc Natl Acad Sci USA 115:E4284–E4293. doi:10.1073/pnas.1807275115 29666229 PMC5939072

[31] Quigley CTC, Capistrant-Fossa KA, Morrison HG, Johnson LE, Morozov A, Hertzberg VS, Brawley SH. 2020. Bacterial communities show algal host (Fucus spp.)/zone differentiation across the stress gradient of the intertidal zone. Front Microbiol 11:563118. doi:10.3389/fmicb.2020.563118 33072025 PMC7541829

[32] Weigel BL, Pfister CA. 2021. Oxygen metabolism shapes microbial settlement on photosynthetic kelp blades compared to artificial kelp substrates. Environ Microbiol Rep 13:176–184. doi:10.1111/1758-2229.12923 33372322

[33] Koteska D, Marter P, Huang S, Pradella S, Petersen J, Schulz S. 2023. Volatiles of the apicomplexan alga Chromera velia and associated bacteria. Chembiochem 24:e202200530. doi:10.1002/cbic.202200530 36416092 PMC10107727

[34] Orsi WD, Smith JM, Liu S, Liu Z, Sakamoto CM, Wilken S, Poirier C, Richards TA, Keeling PJ, Worden AZ, Santoro AE. 2016. Diverse, uncultivated bacteria and archaea underlying the cycling of dissolved protein in the ocean. ISME J 10:2158–2173. doi:10.1038/ismej.2016.20 26953597 PMC4989311

[35] Xue J-H, Zhang B-N, Zhang F, Liu Y-Y, Wu W-J, Wu Z-M, Si Y, Yang P-X, Xing X, Zhao L-H. 2022. Comparative genomic analysis of the genus Marinomonas and taxonomic study of Marinomonas algarum sp. nov., isolated from red algae Gelidium amansii. Arch Microbiol 204:586. doi:10.1007/s00203-022-03215-y 36048288

[36] Loiko N, Islam MN. 2024. Plant–soil microbial interaction: differential adaptations of beneficial vs. pathogenic bacterial and fungal communities to climate-induced drought. Agronomy 14:1949. doi:10.3390/agronomy14091949

[37] Kang E, Li Y, Zhang X, Yan Z, Zhang W, Zhang K, Yan L, Wu H, Li M, Niu Y, Yang A, Wang J, Kang X. 2022. Extreme drought decreases soil heterotrophic respiration but not methane flux by modifying the abundance of soil microbial functional groups in alpine peatland. CATENA 212:106043. doi:10.1016/j.catena.2022.106043

[38] Saha M, Dittami SM, Chan CX, Raina J, Stock W, Ghaderiardakani F, Valathuparambil Baby John AM, Corr S, Schleyer G, Todd J, Cardini U, Bengtsson MM, Prado S, Skillings D, Sonnenschein EC, Engelen AH, Wang G, Wichard T, Brodie J, Leblanc C, Egan S. 2024. Progress and future directions for seaweed holobiont research. New Phytologist 244:364–376. doi:10.1111/nph.20018 39137959

[39] Mahajan M, Jethwani P, Mootapally C. 2025. Seaweed-Bacteria Interaction, Molecular Mechanism and Biotechnological Applications, p 393–424. In Biotechnological Interventions to Aid Commercial Seaweed Farming. Springer Nature Singapore, Singapore.

[40] Kaya C, Uğurlar F. 2024. Glutathione‐induced hydrogen sulfide enhances drought tolerance in sweet pepper (Capsicum annuum L.). Food Energy Secur 13:e559. doi:10.1002/fes3.559

[41] Kuramshina ZM, Khairullin RM. 2023. Endophytic strains of Bacillus subtilis promote drought resistance of plants. Russ J Plant Physiol 70:45. doi:10.1134/S1021443722603172

[42] Yu Q, Sun W, Gao H. 2021. Thiosulfate oxidation in sulfur-reducing Shewanella oneidensis and its unexpected influences on the cytochrome c content. Environ Microbiol 23:7056–7072. doi:10.1111/1462-2920.15807 34664382

[43] Sorokin DY, Rainey FA, Webb RI, Fuerst JA. 2015. Sulfitobacter. In Trujillo ME, Dedysh S, DeVos P (ed), Bergey’s Manual of Systematics of Archaea and Bacteria

[44] Iciek M, Włodek L. 2001. Biosynthesis and biological properties of compounds containing highly reactive, reduced sulfane sulfur. Pol J Pharmacol 53:215–225. https://europepmc.org/article/MED/11785922. 11785922

[45] Han M-L, Lv Q-Y, Zhang J, Wang T, Zhang C-X, Tan R-J, Wang Y-L, Zhong L-Y, Gao Y-Q, Chao Z-F, Li Q-Q, Chen G-Y, Shi Z, Lin H-X, Chao D-Y. 2022. Decreasing nitrogen assimilation under drought stress by suppressing DST-mediated activation of Nitrate Reductase 1.2 in rice. Mol Plant 15:167–178. doi:10.1016/j.molp.2021.09.005 34530166

[46] Wen J, Shi J, Meng M, Xu K, Xu Y, Ji D, Wang W, Xie C. 2025. Metabolic responses of Pyropia haitanensis to dehydration-rehydration cycles revealed by metabolomics. Mar Drugs 23:203. doi:10.3390/md23050203 40422793 PMC12113544

[47] Yanmin Y, Fuli LIU, Zhourui L, Pengyan Z, Yi LIU, Yanxin Z, Haining Z. 2023. Study on the physiological and biochemical influence of Sargassum thunbergii under dehydration. Progress in Fishery Sciences 44:149–160.

[48] Wang Z, Lu C, Chen J, Luo Q, Yang R, Gu D, Wang T, Zhang P, Chen H. 2022. Physiological and multi-omics responses of Neoporphyra haitanensis to dehydration-rehydration cycles. BMC Plant Biol 22:168. doi:10.1186/s12870-022-03547-3 35369869 PMC8978406

[49] Wang W, Ge Q, Wen J, Zhang H, Guo Y, Li Z, Xu Y, Ji D, Chen C, Guo L, Xu M, Shi C, Fan G, Xie C. 2024. Horizontal gene transfer and symbiotic microorganisms regulate the adaptive evolution of intertidal algae, Porphyra sense lato. Commun Biol 7:976. doi:10.1038/s42003-024-06663-y 39128935 PMC11317521

[50] Chen H, Chu J-C, Chen J, Luo Q, Wang H, Lu R, Zhu Z, Yuan G, Yi X, Mao Y, Lu C, Wang Z, Gu D, Jin Z, Zhang C, Weng Z, Li S, Yan X, Yang R. 2022. Insights into the Ancient adaptation to intertidal environments by red algae based on a genomic and multiomics investigation of Neoporphyra haitanensis. Mol Biol Evol 39:msab315. doi:10.1093/molbev/msab315 34730826 PMC8752119

[51] Chen J, Zang Y, Yang Z, Qu T, Sun T, Liang S, Zhu M, Wang Y, Tang X. 2022. Composition and functional diversity of epiphytic bacterial and fungal communities on marine macrophytes in an intertidal zone. Front Microbiol 13:839465. doi:10.3389/fmicb.2022.839465 35369473 PMC8972133

[52] Edgar RC. 2013. UPARSE: highly accurate OTU sequences from microbial amplicon reads. Nat Methods 10:996–998. doi:10.1038/nmeth.2604 23955772

